# The Hospital Egg

**Published:** 1915-07-31

**Authors:** 


### The Hospital Egg.

*To the Editor of* The Hospital.

Sir,—The interesting correspondence on this subject in The Hospital is open, it seems to me, to the criticism that the practical issue is not, strictly, that between the contract system of purchasing eggs and the alternative one of pickling eggs in summer. This may be the case under ordinary circumstances, but, as things are, some of us are wondering whether contractors, even with the best intentions, will be able to carry out their contracts, or can be held to them if difficulties occur. Some hospitals have abandoned the contract system for this reason; and for those who usually resort to it, the advisability of doing so under present circumstances is not a plain issue.—Yours faithfully, Hospital.

